# Values are not enough: qualitative study identifying critical elements for prioritization of health equity in health systems

**DOI:** 10.1186/s12939-020-01276-3

**Published:** 2020-09-15

**Authors:** Thea van Roode, Bernadette M. Pauly, Lenora Marcellus, Heather Wilson Strosher, Sana Shahram, Phuc Dang, Alex Kent, Marjorie MacDonald

**Affiliations:** 1 Canadian Institute for Substance Use Research, University of Victoria, PO Box 1700 STN CSC, Victoria, BC V8W 2Y2 Canada; 2 School of Nursing, University of Victoria, PO Box 1700 STN CSC, Victoria, BC Canada; 3 Faculty of Health and Social Development, University of British Columbia, 1147 Research Road, Okanagan, Kelowna, BC V1V 1V7 Canada

**Keywords:** Health equity, Public health systems research, Priority, Complexity, Social justice, Health inequities

## Abstract

**Background:**

Health system policies and programs that reduce health inequities and improve health outcomes are essential to address unjust social gradients in health. Prioritization of health equity is fundamental to addressing health inequities but challenging to enact in health systems. Strategies are needed to support effective prioritization of health equity.

**Methods:**

Following provincial policy recommendations to apply a health equity lens in all public health programs, we examined health equity prioritization within British Columbia health authorities during early implementation. We conducted semi-structured qualitative interviews and focus groups with 55 senior executives, public health directors, regional directors, and medical health officers from six health authorities and the Ministry of Health. We used an inductive constant comparative approach to analysis guided by complexity theory to determine critical elements for prioritization.

**Results:**

We identified seven critical elements necessary for two fundamental shifts within health systems. 1) Prioritization through informal organization includes creating a systems value for health equity and engaging health equity champions. 2) Prioritization through formal organization requires explicit naming of health equity as a priority, designating resources for health equity, requiring health equity in decision making, building capacity and competency, and coordinating a comprehensive approach across levels of the health system and government.

**Conclusions:**

Although creating a shared value for health equity is essential, health equity - underpinned by social justice - needs to be embedded at the structural level to support effective prioritization. Prioritization within government and ministries is necessary to facilitate prioritization at other levels. All levels within health systems should be accountable for explicitly including health equity in strategic plans and goals. Dedicated resources are needed for health equity initiatives including adequate resourcing of public health infrastructure, training, and hiring of staff with equity expertise to develop competencies and system capacity.

## Background

The health equity gap is well recognized, and there is global recognition of the need to reduce health inequities between and within countries [1, 2]. Health *inequities* are unfair and avoidable systematic differences in health that disadvantage some groups more than others in terms of health outcomes and opportunities [3, 4]. Whitehead and Dahlgren state that “equity in health implies that ideally everyone could attain their full health potential and that no one should be disadvantaged from achieving this potential because of their social position or other socially determined circumstance (page 5)” [3]. Thus, attending to health equity includes the dual goal of aiming to improve health outcomes for the entire population, while striving to reduce existing health inequities [3–5].

Health systems have a fundamental role in addressing health equity; however, better understanding of strategies that help reorient health systems towards health equity is required [6–9]. One critical starting point is through effective prioritization of health equity within health systems, policies and programs. However, creating change is inherently challenging in complex health systems that are nonlinear, dynamic, and evolving, and sensitive to the unique individuals, contexts and circumstances within that system [10–12]. Although enactment of a system-wide policy or program can be an essential first step in prioritization, whether an effective system response emerges from this will depend on the actions of those within the system and other elements that support this prioritization.

Studies that explicitly consider prioritization of health equity within health systems are limited. However, studies considering factors that support health equity action more generally highlight the role of public health, supportive legislation, leadership, values and understanding, resources, capacity and infrastructure [13–19]. In their organizational framework for public health equity action, Cohen et al. stressed the importance of internal and external organizational values and commitments to health equity as evidenced by supportive resources and infrastructure [17]. Additionally, Marmot noted the need for strong leadership and supportive legislation from government health ministries to aid in prioritization across public sectors [15]. A Canadian study investigating health equity action within public health units noted that health equity prioritization within the organization was critical to support health equity initiatives [16]. The authors noted that gaps existed between decisions around policy at one level and implementation plans to support the policy at other levels. A study investigating health equity action within a subset of public health departments in the United States also highlighted the importance of health equity as a priority [13]. They considered evidence of prioritization to include health equity initiatives, strategic plans, creation of equity positions, assessment measures, and collaboration with outside organizations to facilitate health equity work. This study also found that funding and limited training and guidance were significant barriers to health equity work. This highlights that different elements are needed to embed health equity as a priority within health systems.

In British Columbia (BC), Canada, there have been efforts to prioritize health equity through policy changes at the provincial level as part of systemic public health renewal [20–22]. These include health equity considerations in two critical documents that endorsed the application of a health equity lens: the Framework for Core Functions in Public Health and the Guiding Framework for Public Health [20–22]. At the time of this research, there were five regional health authorities, a provincial health authority, a newly introduced First Nations Health Authority (established in 2013), and the Ministry of Health. Regional health authorities were charged with applying a health equity lens to all public health programs. These directives provide an example of efforts to prioritize health equity within a complex system through the enactment of policy changes that need to be taken up and prioritized across the health system. The Equity Lens in Public Health Program of Research (ELPH) was designed to study this implementation of a health equity lens within BC health authorities, during this time of public health renewal and system change [23]. Although little is known about how health equity was being prioritized in the early implementation period of a health equity lens following these directives, prior analysis highlighted a lack of understanding of what constituted this lens and how to apply it [24].

We sought to explore what is needed to support effective prioritization of health equity within complex health systems, from the perspective of senior leaders who were decision makers within that system. As part of the ELPH program of research, we undertook a baseline qualitative study to examine how health equity was being prioritized within BC health authorities during this early implementation period of a health equity lens. Specifically, we aimed to determine critical elements to support effective prioritization within complex health systems, guided by complexity theory.

## Methods

This study is part of The Equity Lens in Public Health (ELPH) program of research that commenced in 2011 to study the application of a health equity lens in BC health authorities [23]. As part of the broader program of research, we conducted a qualitative analysis ‘Assessing Health Equity Priorities’, which included a baseline examination of senior leaders’ perspectives on prioritization of health equity in the BC health system in 2013/2014. Our collaborative research team included public health systems leaders from Fraser Health Authority, Interior Health Authority, Island Health Authority, Northern Health Authority, and Vancouver Coastal Health Authority, the Provincial Health Services Authority, and the Ministry of Health. Ethical approval for this study (H11–03359) was provided by the University of Victoria, the University of British Columbia, the University of Saskatchewan, Fraser Health Authority, Interior Health Authority, Island Health Authority, Northern Health Authority, and Vancouver Coastal Health Authority research ethics review boards.

We used a participatory process in which health authority partners and academic researchers worked collaboratively throughout the entire research process from developing the research questions, through planning the research design, to analyzing and interpreting the findings [25, 26]. Such participatory approaches are reflexive, dialectic process to better align research and theory with practice, and include stakeholders in knowledge generation that is relevant and useful; participatory approaches are aligned with integrated knowledge translation and exchange processes. We developed working groups and held meetings with partners for feedback for each stage of the research process including the development of the research questions, planning the research design, developing the interview guides, and analyzing and interpreting the data. This contributed to relevance and rigour of the research.

The ELPH program of research employs complexity theory, intersectionality, and critical social justice theories as theoretical foundations for research [23, 24]. In this study, we used complexity theory to guide our analytical process about what is needed for effective prioritization within complex health systems. We drew on Walby’s framework for theorizing about the intersections of social relations and institutional structures and the implications of this for supporting system responses to increase prioritization of health equity at multiple levels of the health system [27–29]. Within this understanding of complexity, we considered the implementation of a health equity lens to be an event inserted into the health system, and examined the resulting responses within various programs and levels of the system [10, 12, 30]. We conceptualized health systems as complex adaptive systems, in which system responses to enactment of a policy will depend on the multiple interactions and relationships that occur in that system as a result of that policy [10, 11, 30–32]. Thus, while change within a system will be influenced by formal organizational documents and policies, the system response that emerges will also reflect the informal organization that emerges through created structures, groups and processes [10, 12]. Anderson highlights the importance of understanding both these formal and informal aspects of a system, and the interactions between them, to gain insight into the way a system is functioning as a whole as well as to understand the specific system properties that influence change [10]. We aimed to understand how these formal and informal aspects, and their interactions, vary within different parts of the system, and the potential impact of this on the system response. This included identifying critical elements that facilitate prioritization of health equity within the health system, and the relationships among these elements.

We undertook an inductive qualitative analysis examining perspectives of senior leaders from within the BC health system. We conducted semi-structured qualitative interviews and focus groups with these senior leaders including senior executives, public health directors, regional directors, and medical health officers identified as leaders and decision makers within their organizations and portfolios. The collaborative research team developed a semi-structured qualitative interview guide to seek perspectives on how health equity was being prioritized within the health system, and explore potential barriers and facilitators for prioritization. This was used for both the interviews and focus groups.

We used purposive sampling which aims to select participants that have good knowledge of the area under investigation [33]. Health authority partners on the research team invited all senior leaders within their health authorities through an email invite outlining the purpose of the study and provided information on participation. The principal investigators, who were experienced interviewers and well versed in health equity and health systems services research, conducted the interviews and focus groups. Participation was voluntary and the interviewers obtained written informed consent from each participant prior to beginning the interview. All interviews were audio-recorded and transcribed verbatim by a research associate. Identifying information was removed and cleaned transcripts were stored on a central shared drive at the university with restricted access.

### Analysis

We collected data from January 2013 to July 2014. We used NVIVO 10 to code and analyze focus groups and interviews. We used a constant comparative approach to review the data and inductively develop a coding framework that examined information related to prioritization of health equity [23, 34–36]. This method involves detailed coding to develop concepts and relationships among the data by comparing incident-to-incident, incident-to-concept, and concept-to-concept to advance the analysis to higher levels of abstraction [37]. Three research associates coded all interviews into this framework. Initial results were shared with the health authority partners who provided feedback. The principal investigators and a fourth research associate then inductively derived themes that emerged from the constant comparative coding around prioritization within each organization, as well as barriers and facilitators to prioritization. We then derived critical elements needed for prioritization from these themes guided by complexity theory. We considered differences in these elements across organizations to understand variability and the extent to which they reflected common systemic elements. We identified critical elements for prioritization and examined the interrelationships among these elements, as well as congruence or divergence. We then grouped these into two overarching themes that provide insight into the informal and formal supports needed for prioritization. All authors including those involved in interviewing, initial coding and later analysis reviewed the analysis and interpretation.

## Results

There were 55 senior leaders who participated in 12 individual interviews and 14 focus groups. Approximately half of the sample were men, and half were women. Over half the sample were aged 46–60 years, and the majority had been in their current positions for 1–5 years and had more than 10 years’ experience working in public health overall. The sample had high levels of education with the majority holding an undergraduate university degree or higher degree. Overall, there was good representation across gender, age, years in position, and years in public health to ensure a variety of perspectives. Characteristics of the sample are given in Table 1.

**Table 1:** Characteristics of the sample (*N* = 55)

Characteristic	Number (Percent) N (%)
**Gender**	
Men	29 (52.7)
Women	26 (47.3)
**Age in Years**^a^	
≤ 45	13 (26.0)
46–60	28 (56.0)
≥ 61	9 (18.0)
**Years in Position**	
< 1	4 (7.3)
1–5	35 (63.6)
6–10	9 (16.4)
11–20	5 (9.1)
> 20	2 (3.6)
**Years in Public Health**^a^	
≤ 10	10 (20.4)
11–20	19 (38.8)
≥ 21	20 (40.8)
**Highest Educational Qualification**^a^	
Bachelor or Masters Degree	27 (50.0)
PhD	4 (7,4)
Medical Degree^b^	20 (37.0)
Other	3 (5.6)

^a^Does not sum to 55 due to missing data

^b^Includes Doctor of Medicine/ Fellow of the Royal College of Physicians of Canada/Fellow of the Australian Faculty of Public Health Medicine

We identified seven critical elements necessary to support prioritization of health equity within health systems that were grouped into two overarching themes: 1) Prioritization through informal organization; and 2) Prioritization through formal organization.

### Prioritization through informal organization

We identified two elements of the informal organization that are necessary to support prioritization of health equity from the perspective of senior leaders: 1) creating a systems value for health equity, and 2) engaging health equity champions.

#### Creating a systems value for health equity: “Equity’s a good thing” (INTV 704)

Creating a systems value for health equity was identified as a critical element to support prioritization of health equity within health systems. In this study, we heard that health equity was generally viewed as a value within the health system, but that the strength of this value varied. Participants deemed health equity a core value for public health leaders (e.g. medical health officers and directors within public health and population health). One participant noted that *“from the senior leadership and the medical health officers, it would be their reason for working.” (INTV 505).* This strong value placed on health equity within public health supported prioritization. This was evidenced by statements from public health leaders that reducing inequities was “*their most important goal” (FG 302)* or that health equity was a “*major preoccupation. We’re constantly looking at a variety of ways of bringing it in” (INTV 307).*

However, the extent to which health equity was held as a value in other parts of the health system was variable, and noted as a barrier to prioritization. Participants noted that for many within public health and the health system, health equity was consistent with professional values even when health equity was not explicitly recognized as a value. As this participant observed, a challenge is finding the keys to ‘unlock’ this value:

*“I think it’s back to why most people are working- I think most people, if you really spoken, why they’re working in health, probably one of their core values is around health equity, so, it’s a matter of getting the key to unlock that. I think if we’re going at them and it’s too theoretical, it’s easy to dismiss” (INTV 607).*

As this quote illustrates, values related to health equity align with the values of many who work in health. However, as stated, helping people to identify that their values align relies on understanding health equity concepts which can be challenging.

Further, participants indicated that while health equity may be a value for senior executives outside of public health within a health authority, this did not mean it was necessarily a priority. As one participant noted: “*for the medical health officers, it is a priority. But for us it’s largely a priority in our roles outside of the health authority” (FG 104)*. Thus, while some health equity work occurs through work outside the health authority, the degree to which health equity was a priority for senior executives within their roles within the health authority was inconsistent.

Within the wider health system, including the provincial government, we heard that values related to health equity were shifting, especially in relation to Indigenous peoples’ health. One participant highlighted the importance of historical and political barriers to addressing health inequities in systems in which there is no clear value or prioritization of health equity as evidenced by lack of investments:

“*We haven’t necessarily seen investment from a resource or dollar perspective, but it trickles in a direction that, in the past, … if we went back a decade here, there was a lot more acrimony between the provinces and the federal government when it came to looking at First Nation’s health issues. And provincial governments were less likely to even permit or allow for a provision of services or supports to First Nations, to reserves settings” (INTV103).*

Thus, it is critical to assess where health equity is being held as a value, and aim to increase this value where it is limited or lacking to support prioritization. In this study, we heard that while health equity was a value for public health, it was largely not on the agenda outside of public health. Moreover, value for health equity was hindered by lack of understanding of health equity concepts. This highlights the importance of having a *systems* value for health equity to support health equity prioritization, facilitated by understanding of health equity concepts as being rooted in systemic injustices.

#### Engaging health equity champions: “So …what’s made it a priority to health authority? Well, me” (INTV 407)

Health equity champions were identified as key facilitators for increasing value for health equity and prioritization where systemic prioritization of health equity is not well established or supported. Participants recognized the importance of individual leader’s efforts to move the health equity agenda forward when asked about facilitators for prioritization. Participants identified some senior leaders within the regional health authorities as key champions working to increase the relative importance of health equity work. We heard that these champions worked to create awareness of health equity issues within their respective health authority, make health equity concepts more accessible for staff, and bring in staff with health equity expertise. Participants specifically identified medical health officers (MHOs) as important health equity champions who were able to help bring attention to health equity within their roles on committees or executive tables. One participant from a regional health authority indicated they had attempted to champion health equity at the level of local government:

*“So … this is a personal thing. My colleagues don’t engage in the same level of dialogue with local governments that I do, but I actually go around knocking on local government doors and presenting some of the differences in health status that they either suffer from or benefit from, so they are actually aware themselves” (INTV 103).*

At the provincial level within the Ministry of Health, participants noted some ministers were operating as champions motivated by their value for health equity: “*there are some examples of ministers taking a stand on a position. … and I think that does emanate from their values and something they think they can get a hold of and move forward, and it has some legs against it” (FG 702).* Another participant noted individual clinicians with a strong value for health equity acting as champions, attempting to integrate health equity into their programs from the ground up.

Here, we heard that champions emerged informally from their strong personal value for health equity, often rooted in public health values and roles. Further, participants highlighted the need for champions to operate across all parts of the regional health system and government. As noted, this can create initial momentum for health equity work that can increase awareness, value and competency for health equity, and its relative priority.

### Prioritization through formal organization

We identified five critical elements to embed health equity as a priority in the formal organization from the perspective of senior leaders: 1) explicitly naming health equity as a priority; 2) requiring health equity in decision-making 3) designating resources for health equity; 4) building capacity and competency for health equity; and 5) coordinating a comprehensive approach.

#### Explicitly naming health equity as a priority: “If we went back to the goals, objectives, strategic plans and what not, equity is kind of given short shrift” (INTV 103)

Explicit naming of health equity as a priority was identified as a critical element for overall prioritization within the health system. We heard that health equity was often not explicitly named as an organizational priority in goals and strategic plans, and that lack of “*deliberate application*” *(INTV 307)* of health equity was a barrier to prioritization. In referring to a strategic plan, one participant stated that “*I’ve kind of looked at the whole thing and I just don’t see equity anywhere in this, you know, twenty-five page document” (FG 403).* Similarly, another participant noted: “*our actions are not congruent with what we should be doing from a priority perspective for health equity. … if you look at the strategic plan, reducing inequities is kind of couched and buried in there. It’s not a major priority” (FG 102*). One participant noted that not explicitly naming health equity as an organizational priority created barriers for prioritization: “*the fact that it’s just not really shining as an organizational imperative creates some barriers in itself because we feel like it’s off the corner of our desk” (FG 403).*

When asked about prioritization of health equity, several participants implicitly linked health equity goals with stated health system priorities. These included priorities for primary care, goals for improved health and wellness, strategies such as integrated accessible health services, and initiatives targeting particular populations and aiming to improve access. For example, one participant noted health equity prioritization through *“a partnering with communities endeavor across the organization, this is an organizational priority. It’s part of our integrated accessible health services priority” (INTV 505).* Another participant linked health equity prioritization to primary care but noted this was implicit:

*“I think certainly as a priority, is to give good service to the person in the family. And that’s why the whole primary care model is being used, and that’s really what it’s about. … doing that well naturally deals with the inequity problem, but I don’t think it’s like, we’re doing this to deal with the inequity problem” (FG 504).*

These attempts to link health equity prioritization to formal priorities reinforces the importance of explicit naming of health equity as a priority.

Participants identified lack of explicit prioritization of health equity by the Ministry of Health as an issue that had implications for prioritization within their health authority. One participant clearly articulated the need for ministry prioritization of health equity to facilitate prioritization within their organization: “*equity and inequity is not embedded in our health authority whatsoever. It is not espoused or embedded at the highest levels. That’s probably because it’s not embedded or espoused in the Ministry who tells the health authority [what] to do*” (*FG 301)*. This participant was specifically speaking to the need for explicit naming of health equity as a priority in mandate letters from the Ministry of Health as a means of giving it priority in service plans and strategic plans.

In this study, we heard that reliance on implicit individual prioritization of health equity was a barrier to prioritization of health equity within the health system. The above illustrates the need for explicit prioritization of health equity across all levels of the health system in mandates, and service and strategic plans. This is required in order to translate health equity into a relative priority at all levels of the health system so it can be operationalized into programs and policies.

#### Requiring health equity in decision making: “Ultimately at the end of the day, the question is whether equity is incorporated in” (INTV 103)

Mechanisms to include health equity in decision making were identified as a critical element for embedding health equity as a priority into health systems. Within the regional health authorities, we heard that such requirements and mechanisms were variable and often missing: “*there isn’t an explicit mechanism, and I can’t think of even one today, that requires the board or the senior executive or decision makers, to put equity as an integral part of their decision process” (INTV 103).* Participants indicated this can hinder the ability to prioritize health equity despite the desire to do so.

In contrast, where such mechanisms do exist, participants identified that they can serve as a facilitator for prioritization. Participants gave examples of such mechanisms such as one health authority governance board prioritizing and resourcing health equity across the health authority with “*a requirement for the other twenty-one programs to work with Population Health to put equity into their planning framework” (INTV 201).* Other examples from within public health included health equity as a key principle for planning, or taking health equity into account when making decisions or allocating resources.

Participants also highlighted the importance of representation and inclusion of communities and those impacted by health inequities in decision making processes as a mechanism to facilitate prioritization. This participant indicated the importance of these roles when asked about facilitators for prioritization:

*“I would say ten years ago we- [health authority], … actually had very visible things to point to say, ‘This is a priority for us.’… we had membership on the board with designated seats for Aboriginal populations, people with disabilities, people with mental health and addictions issues. So, you know, there was really sort of very visible communication that we’re trying to represent all of that. And we have- we’ve prioritized it enough to have specific roles assigned to focus on those things and to keep us focused on those things. So, you know, I wouldn’t say we have- we express that perspective on equity and diversity and the diversity of needs, and needing to level playing fields and that sort of thing. We don’t have that kind- because we don’t have those roles anymore” (INTV 307).*

In this scenario, this participant noted that these roles no longer existed as there had been a shift towards becoming more “*business and process focused*” (INTV 307). This highlights a need for representation of communities and groups that experience health inequities to have a voice at the decision-making level of the organization. In addition to this, participants stated the importance of the inclusion of public health and medical health officers at the executive table and in the decision making process as a facilitator to prioritize health equity. As this participant stated: “*having the medical health officer at the executive table and at the board meetings is helpful. Management teams all have a public health presence on their teams. Public Health being actively engaged in the overall priorities of the organization, not separate, I think helps that way” (INTV 505).*

Thus, we heard that it is essential that there are mechanisms that require health equity to be factored into planning processes and resource allocation. Further, participants highlighted one such mechanism is to ensure communities, groups impacted by inequities, and those with health equity values and mandates are represented at executive tables and in decision making. With regard to the latter, participants noted that medical health officers and public health leaders held strong values of health equity, and thus should be included to promote the systematic consideration of health equity in the decision-making process.

#### Designating resources for health equity: “We’re not communicating to the public we really believe these things and we’re putting our money where our mouth is” (INTV 307)

Another essential component of prioritization identified was allocating adequate resources for addressing health equity. In some health authorities, participants reported that a lack of dedicated resources severely limited prioritization. As this participant stated: “*our ability to prioritize, develop a lens around prioritizing resources and allocation, isn’t there, I would say bluntly” (INTV 201).* Despite these challenges, we heard that participants were attempting to prioritize resources for health equity where possible. As one participant stated: “*so from my perspective, I’d like to believe that I, and I can only speak for myself, at least keep the concept of equity on my plate when I am trying to divvy up what limited resource I have” (INTV 103).* This highlights that prioritization relied on individual leaders’ commitment to health equity but was not necessarily widespread.

As well, we heard that prioritizing resources for health equity was a challenge due to limited resources overall and competing priorities, such as for acute care. One participant commented on a discrepancy between perceived values of the health authority and concrete prioritization through adequate funding to the public health sector: “*they [health authority] talk inequity, and they acknowledge inequity--but when it comes to the bottom line, our budget has been pillaged for the benefit of the acute care sector” (FG 104).*

We heard that increases to budgets for population health or specifically for improving Indigenous health, were perceived to be consistent with a commitment to prioritizing resources for health equity: “*we got a significant increase in the budget for Population Health, at a time when previously we’d not spent the budget that we had. So I think that spoke to the commitment that the exec has. It’s shared by the board*” *INTV 201).* This highlights that investments in areas that carry out health equity work, or where burden of heath inequities are high, are needed to support prioritization.

In this study, with limited exceptions, participants indicated there was little specific resourcing to support the prioritization of health equity initiatives due to competing and dominant priorities within the health system. This emphasizes the need for high level investments to support health equity prioritization, including investments in areas such as public health, Indigenous health, and mental health and substance use. Further, dedicated resources are required to support necessary training to develop health equity capacity and competencies that are another critical element to support prioritization.

#### Building capacity and competency for health equity: ‘There’s a nod towards it, but I don’t think it’s understood” (FG 104)

A fourth critical element for effective prioritization of health equity identified was ensuring adequate system capacity and competencies for health equity. This includes prioritizing necessary training and leadership to create this system capacity. We heard there were attempts to prioritize health equity across the health system without adequate guidance, necessary system capacity or competencies to support this. This participant discussed this lack of guidance when asked about barriers to prioritization:

*“If we went back to the public health core programs you know when this, I shouldn’t say started then but you know people were talking ah, inequity lenses and trying to increase our focus of attention in such a fashion to reduce inequities, there were real challenges about how that or what that looked like” (INTV 103).*

Without clear guidelines as part of this early implementation process, participants indicated that attempts to determine what constituted a health equity lens and how to implement it were occurring at all levels. This participant noted that there was significant variation in capacity that impacted prioritization: “*The depth of understanding and then application of that is variable from program to program and leader to leader and…front line to front line” (INTV 307).* Participants indicated that those within public health, Indigenous health, and mental health and addictions were better versed in health equity and therefore more readily able to engage in health equity work than in other areas of the system such as acute care or primary care.

In some cases, participants indicated that senior leadership responsible for prioritization lacked necessary competency: “*Well, but it’s not a requirement of our senior executive or senior administration that they actually be knowledgeable about equity, that they incorporate it into what they do. And so we’ve got people there that are still based upon what they used to do and equity …was not part of the conversation*” *(INTV 103).* We heard that this lack of general understanding about health equity concepts was missing at all levels of the health system, including at the ministry level.

This lack of health equity competencies contributed to an overall lack of system capacity that was evident within the health system. We heard that there were few health equity roles to support the development of competencies and capacity. One participant stated that in their health authority they were relying on one or two designated people within public health to be responsible for health equity work: “*There are one and a bit people that mainly do equity type issues and have a portfolio for doing things” (INTV 201).* Another indicated that prior roles that focused on such issues no longer existed. We also heard that competing pressures limited system capacity for health equity work.

Within this context, we heard examples of initial attempts within the health system to address this lack of competency and capacity. One participant discussed informal attempts by leadership to improve understanding and competency for health equity work to support wider prioritization. They also noted the challenges inherent in this:

*“I’m trying to move the focus upstream- always challenging in that kind of an environment. But I think there’s a commitment in the organization trying to do that. [Name] had a really nice way of encouraging to do that, … [they] would talk about move one step upstream. “You work in an ICU unit, what’s the thing that you could do that’s upstream from where you are now?” I think that’s an easier thing to ask of people than to ask seven thousand employees to think ‘population health’” (INTV 505).*

This highlights that an essential component of prioritization is also to prioritize establishing the necessary guidelines and training that develop health equity capacity and competency. As identified here, engaging strong leadership and developing health equity specific roles can help support this development.

#### Coordinating a comprehensive approach: “You can’t do it without local government, you also can’t do it without the health authority, you’ve got to have everyone there, and headed in the same direction” (INTV 103)

A final element identified was that health equity needs to be embedded as a priority across all areas and levels of the system to be effective. This includes prioritizing health equity within programs and policies, the regional health authority itself, provincial government, and within partnerships with community partners to coordinate a comprehensive and integrated response.

Analysis of participant’s interviews indicated that although there was variation in terms of how extensively health equity was being prioritized across regional health authorities, there were examples of health authorities seeking to build this more comprehensive approach. Participants noted prioritizing initiatives designed to operate across public health, primary care, mental health and addictions, and community and Indigenous partnerships. Participants also highlighted the importance of organizational wide policies related to community and Indigenous partnerships to support prioritization, as well as specialized work “*organized in a way that stimulates the rest of the organization to move upstream” (INTV 505).*

Participants within the health authorities identified health equity prioritization at the Ministry of Health as a potential way to help health equity “permeate into the health authorities” (INTV 307). Furthermore, they noted that where local government was engaged such as at the municipal level, they were a powerful facilitator for health equity prioritization. One participant stated that the local government was actively involved in trying to address health inequities within their communities:

*“And I think the local government’s been a major driver in actually trying to come up with some solutions, more so than the health authority, even though the health authority is involved. So that’s the second step is to then get the information in the hands of the people that will actually drive change” (INTV 103).*

It was noted, however, that changing governments can result in shifting priorities so that support for certain approaches or issues no longer existed. Moreover, we heard that government policies and regulations were experienced as barriers to prioritization if they restricted health authorities in their ability to act. Participants described approaches to health equity at the government level as “piecemeal” rather than fully committed, and potentially without a solid understanding of how to address equity considerations:

*“I think provincially if we look at sort of where government is at, I think there’s awareness of it [health equity], but I think they’re in the same boat as our senior execs, they don’t know quite what to do with it. There’s a lot of piecemeal stuff on it, and so then they go into their whole sort of ‘political beliefs’ and what their party values are and whatever … I think that our government is too business-oriented and it’s… it’s really hard to talk about true equity. But if you talk about access to service and if you talk about standardization of services and health, and all this kind of stuff, there’s a lot of agreement on that. There’s also an agreement on a focus of … rural and remote communities right now; a focus on vulnerable populations- … but, you can start to apply it, but it’s not a comprehensive whole … belief in value and concrete- like it’s not integrated” (INTV 611).*

This supports the importance of prioritization both internally across all levels of the organization, as well as at the periphery with government and through community to be successful in embedding health equity as a priority. As noted above, however, competing values related to a ‘business orientation’ work against prioritization as do values that privilege acute care and biomedicine. As such, a comprehensive approach to prioritizing health equity also requires creating a systems value for health equity. There is a need to invest in a comprehensive, coordinated response that addresses each of the critical elements to successfully increase prioritization of health equity within health systems.

## Discussion

We conducted semi-structured qualitative interviews with senior leaders in regional and provincial health systems during the early stages of attempting to implement a health equity lens in BC health authorities. We aimed to understand critical elements required to support prioritization of health equity within health systems. We identified the importance of elements of both informal and formal organization to support prioritization. There is a need for a systems value for health equity, with champions in every area of the system to increase this value and relative priority for health equity work. However, this value in itself is not sufficient to ensure health equity is prioritized. Health equity must be structurally embedded as priority through explicit naming of health equity in goals, strategic plans, and mandates. Explicit inclusion must be supported by dedicated resources, requirements to include equity in decision making, and development of competencies and capacity for health equity. A comprehensive approach across the health system is required, with attention to these critical elements in every area of the health system, and across levels of government.

Although few studies have explicitly examined what is required to support prioritization of health equity within a health system, the elements identified here align well with factors deemed important to implementation of health equity actions and implementation frameworks [13, 15–17, 19, 38–40]. Participants identified the importance of values for health equity throughout the health system to facilitate prioritization. This aligns with studies that have noted the importance of values for shaping organizational culture and motivating health equity action overall [16, 39, 40]. However, even where a value for health equity exists, this may be limited by lack of understanding as we noted here and competing ideologies [14, 16, 24]. For example, Smith et al., found that senior public health leaders were more comfortable with health equity when it was associated with addressing inequities in access to care and material determinants of health than when it focused on political issues, power imbalances and systemic disadvantage [14]. This points to the importance of a systems value for health equity underpinned by social justice and grounded in recognition of structural causes of inequities [16, 17, 24].

Having health equity champions also emerged as an important element to support prioritization. As noted in this study, health equity champions can have a role in helping to create value and increase relative priority for health equity, as well as support development of health equity competencies within their organizations. Although in this study champions emerged informally as a result of their strong values for health equity, they can also be strategically inserted [40]. Inserting health equity champions with health equity in their portfolio at every level of the health system is a strategy that can help increase recognition of the importance of creating more equitable health systems and drive prioritization [17, 40, 41]. This can be through development of health equity specific positions across the organization or designating those with health equity competencies, such as those in public health, in this role [16, 39, 41]. As well, having those in formal leaderships positions as champions can facilitate prioritization of health equity within formal processes [16, 42].

Our findings align with others recommendations that health equity needs to be explicitly embedded as a priority in the formal organization [15, 16, 40]. This includes the importance of explicit naming of health equity as a priority in formal documents. Participants indicated that prioritization of health equity at the level of ministries and government, with associated mandates, is needed to facilitate prioritization within the health system. These should direct and support organizations to explicitly name health equity in their strategic plans and goals and include strategies for improving health equity [13, 16, 42]. Inclusion of such strategies and guidelines are important to ensure prioritization of health equity actions does not become limited, for example to a focus on targeted initiatives or access issues [39].

Requiring systematic consideration of health equity within decision making is a strategic way to increase prioritization. As noted above, explicit naming of health equity in mandates, strategic plans and goals are critical to help ensure health equity is factored into decision making. Further, our findings support the importance of having those with health equity competencies, such as public health and medical health officers, at decision making tables to facilitate prioritization [39]. Inclusion of those most impacted by health inequities and communities in the decision-making process is fundamental, including throughout program planning stages, to begin to shift power dynamics and increase prioritization [7, 17, 18, 39]. Further, adoption of a health equity lens can be used to support prioritization of health equity within decision making processes [39].

We identified that dedicated resources are needed to support effective prioritization. Limited resources have been highlighted as a barrier to health equity work overall [13]. Dedicated resources have been noted as important to establish relative priority and accountability for health equity where budgets are limited and there are competing priorities [39]. These are needed not only for implementation of health equity initiatives, but also the training and hiring of staff with equity expertise to develop health equity competencies and system capacity [39]. This should be combined with overall investment in the health system and public health infrastructure [7, 39, 40, 43].

We heard that health equity capacity and competencies are required to facilitate effective prioritization. This includes the need for skilled personnel in leadership positions, and the development of health equity positions, across the organization [16, 39]. In this study, participants indicated that one barrier to prioritization was that those in leadership positions sometimes lack necessary health equity competencies. As noted by Dean et al., committed leaders with health equity competencies are required to ensure that policies and resource distribution attend to health equity [42]. In their organizational framework for public health equity action, Cohen et al., stressed the importance of commitment, as evidenced through capacity and competencies, which included personnel with the ability to interpret local data on inequities and advocacy skills [17]. Narain et al., in their study within public health departments in the United States, also considered health equity specific positions to be evidence of prioritization supporting the need for such infrastructure to increase prioritization [13].

Participants stressed the need for a comprehensive approach that attends to these elements across the health system. This is consistent with other studies that have noted the importance of health equity integrated as part of the broader organization rather than limited to specific program areas [16, 18, 39]. Cohen et al., stress the importance of an enabling external environment with government resource allocation that prioritizes equity-based public policies [17]. Such prioritization at the political level is also important to facilitate prioritization within the health system as discussed above, as well as to address determinants of health equity that lie beyond the health sector [15, 43–45].

The evident lack of health equity competencies raises issues around what health equity competencies should entail and how best to develop these. As a fundamental starting place this includes core competencies identified for public health [46, 47]. It further includes knowledge of health equity concepts including recognition of sources of inequities and oppression (colonialism/racism, gender and sex discrimination, ageism, ableism, neoliberalism/capitalism), power and privilege; familiarity with available resources, research, evidence and tools; and what constitutes a health equity lens and strategies to apply it within policies, programs and practices [48, 49]. One strategy to improve competencies within health systems is through curriculum in associated programs [50–53]. Further work is needed to continue to define these competencies and develop effective training resources with sufficient coverage and depth.

This study highlights the potential role of public health in facilitating health equity prioritization as public health was identified here as having strong values and priority for health equity, and a long history of engaging in work that aligns with principles of social justice [17, 39, 54]. This includes engagement in intersectoral action to address structural conditions outside of the health system [39, 55]. However, there may still be considerable variation in the level of capacity and competencies for health equity within public health as in other areas of the system [56]. The Closing the Gap report indicated the importance of policies responsible for health inequities and recognition that social conditions are shaped by the distribution of money, power and resources, aligning with the social justice underpinnings of public health [2]. However, Brassolotto et al. highlighted that public health holds a range of views on the social determinants of health, and understanding that health inequities are structurally produced by policy decisions is not the same as understanding the role that power and privilege play in the production and redress of health inequities [44]. Understanding of health equity concepts may vary within public health, and even public health may have a preference for apolitical views of health equity as noted earlier [14, 56].

The views and values of those across the system (as indicated above) may be influenced by limited resources, competing priorities and systemically embedded ideologies, such as efficiency, individualism, colonialism, and biomedicine common within health systems as well as in governments [8, 43, 57]. Creating a systems value across all levels of the health system and government may be an essential starting point to advocate for necessary structural change that addresses root causes of inequities [45]. Careful consideration of how economic arguments, including costs effectiveness analyses, might be structured to explicitly weigh health equity is a potential strategy for attempting to counter this [58–60]. Development of health equity indicators and surveillance measures, including those related to structural determinants, are needed to demonstrate burden and gradients in health outcomes [61, 62]. Moreover, this highlights the need for formal adoption of a health equity lens at the systems level to anchor all aspects of the health equity agenda, including the critical aspect of prioritization. This must be a comprehensive lens that operates through recognition of and attention to the multiple intersecting sources of oppression and enactment of these within institutional structures that create inequities, as well as recognition of how power and privilege operate within health systems prioritization [24, 62, 63].

Strengths of this study include coverage across five regional health authorities, the Provincial Health Services Authority, and Ministry of Health, to enable comparison across health authorities, and different levels of the system. The study was conducted during a time of complex system change that allowed prioritization of a health equity lens during the early implementation period to be examined. Our collaborative team included health authority partners who were engaged throughout the research process. As there was limited literature with a specific focus on health equity prioritization within health systems, semi-structured qualitative interviews were used to allow ideas that emerged to be explored in depth. Limitations include that a small number of focus groups included both senior executives and members of the program level such as managers in the same interview. This may have limited participant disclosure, particularly for those at the program level if they hold contradictory views to those in senior positions as there are power differentials. Because this study concentrates on the perspective of senior executives, this is less likely to be an issue. However, to limit the impact of this, themes were developed inductively using interviews and focus groups that solely contained senior executives. Information from the mixed focus groups was then used to better understand variation between health authorities within these themes. Moreover, those who did participate may have a greater interest in issues around health equity. This may have made them more aware of initiatives related to health equity within their organizations, or potentially more likely to perceive this to be lacking. As the First Nations Health Authority was newly formed their perspectives were not included in the analysis. Future research is needed to see if there are additional elements that emerge based on the perspectives at the program level.

## Conclusion

Health systems have a role in improving population health and reducing inequities, and must prioritize health equity to address differentials in health and opportunities for health. We sought the perspectives of senior leaders responsible for decision making within British Columbia health authorities to explore how to support prioritization within complex health systems. Effective prioritization of health equity within health systems must take into account competing and dominant values of efficiency, colonialism, individualism and biomedicine, and the lack of understanding of health equity concepts that act as barriers. Creating a core value for health equity throughout the health system is fundamental to counter this, but not sufficient to ensure prioritization. Steps must be taken to build the structural support to formally embed health equity as a priority. This includes recognising the important role of public health, Indigenous health, and other equity oriented program areas in promoting health equity, while striving to embed health equity as a priority broadly across the organization. Dedicated resources are needed to increase the relative priority for health equity, and for health equity roles, training, and initiatives to develop the system capacity and competency necessary to support prioritization that addresses root causes of health inequities. Moreover, prioritization requires involvement from all arenas integrated across the health system. This includes representation of people most impacted by inequities, communities, and those with health equity competencies in decision making processes to ensure relevant and systematic consideration of health equity priorities. There is also a need for engagement from ministries and government to mandate prioritization within health systems, contribute to resourcing, ensure accountability, and coordinate action in other sectors. Future research is needed to develop and evaluate strategies to integrate these elements to increase prioritization in practice. This includes defining health equity competencies and developing adequate training resources, as well as providing guidance on how to apply a systems level health equity lens to embed health equity as a priority.

## Data Availability

Data are not available due to the sensitive and confidential nature of the data.

## Abbreviations

WHO: World Health Organization
BC: British Columbia
ELPH: Equity Lens in Public Health
MHO: Medical Health Officer

## References

[1] Marmot M, Allen J, Bell R, Goldblatt P (2012). Building of the global movement for health equity: from Santiago to Rio and beyond. Lancet.

[2] Marmot M, Friel S, Bell R, Houweling TA, Taylor S, Commission on Social Determinants of Health (2008). Closing the gap in a generation: health equity through action on the social determinants of health. Lancet.

[3] Whitehead M, Dahlgren G (2006). Concepts and principles for tackling social inequities in health: Levelling up Part 1. World Health Organ.

[4] Braveman P (2014). What are health disparities and health equity? We need to be clear. Public Health Rep.

[5] Graham H (2004). Social determinants and their unequal distribution: clarifying policy understandings. Milbank Q.

[6] Östlin P, Schrecker T, Sadana R, Bonnefoy J, Gilson L, Hertzman C (2011). Priorities for research on equity and health: towards an equity-focused health research agenda. PLoS Med.

[7] Gilson L, Lowenson R, Francis V (2007). Challenging inequity through health systems: final report, knowledge network on health systems.

[8] Baum FE (2016). Health systems: how much difference can they make to health inequities?. J Epidemiol Community Health.

[9] Baum FE, Bégin M, Houweling TA, Taylor S (2009). Changes not for the fainthearted: reorienting health care systems toward health equity through action on the social determinants of health. Am J Public Health.

[10] Anderson RA, Crabtree BF, Steele DJ, McDaniel RR (2005). Case study research: the view from complexity science. Qual Health Res.

[11] Cilliers P (2002). Complexity and postmodernism: understanding complex systems: Routledge.

[12] Goldstein J (1999). Emergence as a construct: history and issues. Emergence..

[13] Narain KDC, Zimmerman FJ, Richards J, Fielding JE, Cole BL, Teutsch SM (2019). Making strides toward health equity: the experiences of public health departments. J Public Health Manag Pract.

[14] Smith MJ, Thompson A, Upshur RE (2018). Is ‘health equity’bad for our health? A qualitative empirical ethics study of public health policy-makers’ perspectives. Can J Public Health.

[15] Marmot M, Allen J. Prioritizing health equity. Health All Policies. 2013;63.

[16] McPherson C, Ndumbe-Eyoh S, Betker C, Oickle D, Peroff-Johnston N (2016). Swimming against the tide: a Canadian qualitative study examining the implementation of a province-wide public health initiative to address health equity. Int J Equity Health.

[17] Cohen BE, Schultz A, McGibbon E, VanderPlaat M, Bassett R, GermAnn K (2013). A conceptual framework of organizational capacity for public health equity action (OC-PHEA). Can J Public Health.

[18] Sokol R, Moracco B, Nelson S, Rushing J, Singletary T, Stanley K (2017). How local health departments work towards health equity. Eval Program Plann.

[19] Betancourt JR, Tan-McGrory A, Kenst KS, Phan TH, Lopez L (2017). Organizational change management for health equity: perspectives from the disparities leadership program. Health Aff.

[20] Ministry of Health Services Population Health and Wellness (2005). Public health renewal in British Columbia: an overview of core functions in public health.

[21] Ministry of Health Services Population Health and Wellness (2005). A framework for core functions in public health.

[22] British Columbia Ministry of Health Population Health and Wellness (2013). Promote, protect, prevent: our health begins here. B.C's Guiding Framework for Public Health. Ministry of Health Services, editor. Victoria, BC.

[23] Pauly B, MacDonald M, Hancock T, Martin W, Perkin K (2013). Reducing health inequities: the contribution of core public health services in BC. BMC Public Health.

[24] Pauly B, Shahram SZ, Dang PT, Marcellus L, MacDonald M (2017). Health equity talk: understandings of health equity among health leaders. AIMS Public Health.

[25] Reason P, Bradbury H (2001). Handbook of action research: participative inquiry and practice: Sage.

[26] Kemmis S, McTaggart R, Denzin NK, Lincoln YS (2005). The Sage handbook of qualitative research. Participatory action research: Communicative action and the public sphere.

[27] Walby S, Armstrong J, Strid S (2012). Intersectionality: multiple inequalities in social theory. Socioogy..

[28] Walby S (2009). Theorizing multiple social systems. Globalization and inequalities: complexity and contested modernities.

[29] Walby S (2007). Complexity theory, systems theory, and multiple intersecting social inequalities. Philos Soc Sci.

[30] Hawe P, Shiell A, Riley T (2009). Theorizing interventions as events in systems. Am J Community Psychol.

[31] Leischow S, Milstein B (2006). Systems thinking and modeling for public health practice. Am J Public Health.

[32] Plsek P (2001). Appendix B: Re-designing healthcare with insights from the science of complex adaptive systems. Institute of Medicine Committee on Quality of Health Care in America, editor. Crossing the quality chasm: a new health system for the 21st century.

[33] Polit DF, Beck CT (2008). Nursing research: generating and assessing evidence for nursing practice: Lippincott Williams & Wilkins.

[34] Fram SM (2013). The constant comparative analysis method outside of grounded theory. Qual Rep.

[35] Corbin J, Strauss A (2008). Basics of qualitative research: techniques and procedures for developing grounded theory.

[36] Boeije H (2002). A purposeful approach to the constant comparative method in the analysis of qualitative interviews. Qual Quant.

[37] Glaser B (1978). Theoretical Sensitivity: Advances in the Methodology of Grounded Theory.

[38] Damschroder LJ, Aron DC, Keith RE, Kirsh SR, Alexander JA, Lowery JC (2009). Fostering implementation of health services research findings into practice: a consolidated framework for advancing implementation science. Implement Sci.

[39] National Collaborating Centre for Determinants of Health (2018). Building a culture of equity: 2017 environmental scan.

[40] Simms C (2017). Increasing organizational capacity for health equity work: a literature review for Health Nexus.

[41] BC Centre for Disease Control (2016). Taking action on health equity in environmental public health: five strategies for organizational change.

[42] Dean HD, Roberts GW, Bouye KE, Green Y, McDonald M (2016). Sustaining a focus on health equity at the centers for disease control and prevention through organizational structures and functions. J Public Health Manag Pract.

[43] Baum FE, Laris P, Fisher M, Newman L, MacDougall C (2013). “Never mind the logic, give me the numbers”: former Australian health ministers’ perspectives on the social determinants of health. Soc Sci Med.

[44] Brassolotto J, Raphael D, Baldeo N (2014). Epistemological barriers to addressing the social determinants of health among public health professionals in Ontario, Canada: a qualitative inquiry. Crit Public Health.

[45] Crammond BR, Carey G (2017). Policy change for the social determinants of health: the strange irrelevance of social epidemiology. Evid Policy.

[46] Public Health Agency of Canada (2007). Core competencies for public health in Canada. Ottawa, ON.

[47] National Collaborating Centre for Determinants of Health (2013). Public health speaks: advancing health equity through public health competencies.

[48] National Collaborating Centre for Determinants of Health (2015). Do public health discipline-specific competencies provide guidance for equity-focused practice?.

[49] Public Health Association of British Columbia (2011). Developing health equity competency statements: Public Health Association of British Columbia.

[50] Thornton M, Persaud S. Preparing today's nurses: social determinants of health and nursing education. Online J Issues Nurs. 2018;23(3).

[51] Noriea AH, Redmond N, Weil RA, Curry WA, Peek ME, Willett LL (2017). Development of a multifaceted health disparities curriculum for medical residents. Fam Med.

[52] Talati AN, Lappen JR, Bondurant-Sullivan A, Cossler NJ, Wieczorek M, Gecsi KS (2018). Starting health disparities education during resident orientation: our patients, our community. Obstet Gynecol.

[53] Beavis ASW, Hojjati A, Kassam A, Choudhury D, Fraser M, Masching R (2015). What all students in healthcare training programs should learn to increase health equity: perspectives on postcolonialism and the health of Aboriginal peoples in Canada. BMC Med Educ.

[54] Sadana R, Blas E (2013). What can public health programs do to improve health equity?. Public Health Rep.

[55] Ndumbe-Eyoh S, Moffatt H (2013). Intersectoral action for health equity: a rapid systematic review. BMC Public Health.

[56] National Collaborating Centre for Determinants of Health (2010). Integrating Social Determinants of Health and Health Equity into Canadian Public Health Practice: Environmental Scan 2010.

[57] Farrer L, Marinetti C, Cavaco YK, Costongs C (2015). Advocacy for health equity: a synthesis review. Milbank Q.

[58] Rutstein SE, Price JT, Rosenberg NE, Rennie SM, Biddle AK, Miller WC (2017). Hidden costs: the ethics of cost-effectiveness analyses for health interventions in resource-limited settings. Glob Public Health.

[59] Cookson R, Mirelman AJ, Griffin S, Asaria M, Dawkins B, Norheim OF (2017). Using cost-effectiveness analysis to address health equity concerns. Value Health.

[60] Lal A, Moodie M, Peeters A, Carter R (2018). Inclusion of equity in economic analyses of public health policies: systematic review and future directions. Aust N Z J Public Health.

[61] Penman-Aguilar A, Talih M, Huang D, Moonesinghe R, Bouye K, Beckles G (2016). Measurement of health disparities, health inequities, and social determinants of health to support the advancement of health equity. J Public Health Manag Pract.

[62] Liburd LC, Hall JE, Mpofu JJ, Marshall Williams S, Bouye K, Penman-Aguilar A (2020). Addressing health equity in public health practice: frameworks, promising strategies, and measurement considerations. Annu Rev Public Health.

[63] Pauly B, Shahram SZ, van Roode T, Strosher HW, MacDonald M (2018). Reorienting health systems towards health equity: The Systems Health Equity Lens (SHEL).

